# A258 ROSAI-DORFMAN0-DESTOMBES DISEASE: A RARE CAUSE OF OBSTRUCTIVE JAUNDICE

**DOI:** 10.1093/jcag/gwab049.257

**Published:** 2022-02-21

**Authors:** D Akhtar, M A Donaldson, N H Akhtar, D Owen, S Gan

**Affiliations:** 1 Medicine, The University of British Columbia Faculty of Medicine, Vancouver, BC, Canada; 2 The University of British Columbia Department of Medicine, Vancouver, BC, Canada; 3 School of Medicine, University College Dublin, Dublin, Ireland

## Abstract

**Background:**

Rosai-Dorfman-Destombes Disease (RDD) is rare histiocytic disorder that is most frequently seen in children and young adults. Gastrointestinal involvement is reported in <1% of cases and typically involves the small bowel and colon. Pancreatic and hepatic involvement has been previously reported but is extremely rare.

**Aims:**

To describe a case of obstructive jaundice in the setting of a very rare histiocytic disorder known as RDD.

**Methods:**

Case Report

**Results:**

A 59-year old previously healthy male of Asian descent presented with obstructive jaundice. Initial imaging demonstrated intra and extrahepatic biliary duct dilation with concurrent diffuse enlargement of the pancreas compatible with autoimmune pancreatitis. Endoscopic retrograde cholangiopancreatography (ERCP) was performed with stenting and biopsy. ERCP demonstrated a distal common bile duct stricture with biopsies suggestive of low grade reactive changes and inflammation. Subsequent endoscopic ultrasound (EUS) guided biopsy of the pancreas showed active and chronic inflammation, necrosis, and atrophic pancreatic tissue, with no definitive evidence of autoimmune pancreatitis (Figure 1). Ca-19-9 and IgG4 were normal. He was treated with a course of prednisone and responded well, with repeat CT imaging showing almost complete resolution of the previously demonstrated pancreatic changes. A diagnosis of autoimmune pancreatitis was made. He re-presented 6-months later, however, with fatigue and repeat imaging now displayed lymphadenopathy in the neck, chest, and abdomen, and a bulky pancreatic head with associated hepatomegaly. Lymph node excisional biopsy confirmed the diagnosis of RDD with the presence of scattered histiocytic cells showing emperipolesis with a low number of IgG4 positive cells (Figure 1). The patient was promptly initiated on prednisone and rituximab and has since then had excellent clinical response.

**Conclusions:**

RDD is a rare non-Langerhans cell histiocytosis of unknown etiology that has a prevalence of 1:200 000. RDD clinically presents with painless bilateral cervical lymphadenopathy and can manifest with both nodal and extra nodal involvement. The most common sites of extra nodal disease are the skin and central nervous system, but rarely, can also present with pancreatic involvement. The use of fine needle guided biopsy in diagnosing RDD with extra nodal disease can be limited by low yield, sclerotic tissue, or non-diagnostic findings. For this reason, RDD with pancreatic involvement can masquerade as autoimmune pancreatitis, pancreatic malignancy and IgG4-related disease.This case report raises awareness about RDD with pancreatic and biliary involvement, a rare entity, that can present with obstructive jaundice.

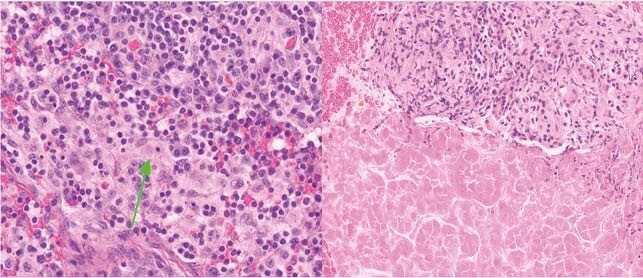

Figure 1: Histological sections of lymph node(left) with hystiocytic cells showing emperipolesis(arrow) and pancreas(right) showing active and chronic inflamation, necrosis and atrophy

**Funding Agencies:**

None

